# Rupture of the Fallopian Tube

**Published:** 1848-04

**Authors:** 


*Sectio Cadaveris.*—On opening the abdomen, a quantity of thin fluid, tinged with blood, flowed out. At the lower part of the abdomen was a large clot, the size of a saucer, and on removing this the left Fallopian tube was found ruptured. The rupture was situated near the junction of the middle and inner third of the tube; the ruptured portion was distended by a mass of fibrin, about the size of a hazel nut, in which, however, no ovum was discoverable; the wall of the distended portion was very thin. The uterus was enlarged, and contained a deposit of decidua.

